# Analytical and Clinical Interference of Sample Hemolysis in Evaluating Blood Biochemical and Endocrine Parameters in Cows

**DOI:** 10.3390/ani14121773

**Published:** 2024-06-12

**Authors:** Dražen Kovačević, Marko Cincović, Mira Majkić, Jovan Spasojević, Radojica Djoković, Sandra Nikolić, Maja Došenović Marinković, Biljana Delić Vujanović, Nemanja Obradović, Ljiljana Anđušić, Aleksandar Čukić, Miloš Petrović, Jože Starič, Jožica Ježek

**Affiliations:** 1 Faculty of Agriculture, University of Novi Sad, Square Dositeja Obradovića 7, 21000 Novi Sad, Serbia; drazen.kovacevic84@gmail.com (D.K.); or marko.cincovic@polj.uns.ac.rs (M.C.); miramajkic@gmail.com (M.M.); jovan.spasojevic1984@yahoo.com (J.S.); neckovs021@gmail.com (S.N.); 2 Faculty of Agronomy, University of Kragujevac, Cara Dušana 34, 32000 Čačak, Serbia; radojicadjokovic@gmail.com (R.D.); petrovic.milos87@yahoo.com (M.P.); 3 Academy of Applied Studies Šabac, Dobropoljska 5, 15000 Šabac, Serbia; m.d.marinkovic@akademijasabac.edu.rs (M.D.M.); b.d.vujanovic@akademijasabac.edu.rs (B.D.V.); 4 Pasteur Institute Novi Sad, Hajduk Veljkova 1, 21000 Novi Sad, Serbia; nemanjanskonzole@gmail.com; 5 Faculty of Agriculture, University of Priština, Kopaonička bb, 38219 Lešak, Serbia; ljiljana.andjusic@pr.ac.rs (L.A.); aleksandar.cukic@pr.ac.rs (A.Č.); 6 Veterinary Faculty, University of Ljubljana, Gerbičeva 60, 1000 Ljubljana, Slovenia; joze.staric@vf.uni-lj.si

**Keywords:** cows, metabolic profile, hemolysis, variability, interference, interferogram

## Abstract

**Simple Summary:**

The metabolic profile implies simultaneous determination of carbohydrate, fat, protein, and mineral metabolism parameters as well as endocrinological parameters in the blood of cows. Blood is exposed to a variety of preanalytical factors during sampling, transport to the laboratory, and laboratory preparation for analysis, which may cause hemolysis of the sample. As hemolysis affects the values of the metabolic profile, the analyzed blood parameters may falsely increase or decrease, and the metabolic status of the cows may be misinterpreted. Preventing hemolysis is important because severe hemolysis requires discarding the sample and resampling, which is very resource-intensive. In this paper, three levels of hemolysis were determined for each blood parameter tested: (a) a hemolysis level that does not affect the values of the parameters and allows the results to be issued without restriction; (b) a hemolysis level that affects the values of the parameters but remains within the acceptable biological variability and permits the results to be issued along with a note in the form of a correction formula; and (c) a hemolysis level at which the obtained values of the parameters or the entire sample must be discarded. The results are presented graphically using interferograms, which can be easily implemented in every laboratory after validation.

**Abstract:**

Hemolysis is a common cause of errors in laboratory tests as it affects blood parameters and leads to a positive or negative bias. This study aims to examine the relationship between the level of hemolysis (expressed as cell-free hemoglobin concentration, g/L) and the variability of metabolic and endocrine parameters and to determine the threshold level of hemolysis that causes an analytically and clinically significant bias for the twenty most frequently examined blood parameters in cows. Paired blood samples of 10 mL each were obtained from 30 cows. One was subjected to mechanical trauma and plasma was extracted directly from the other. Hemolyzed and non-hemolyzed samples from the same animal were mixed to obtain final samples with cell-free hemoglobin concentrations of 0, 1, 2, 4, 6, 8, and 10 g/L. Metabolic and endocrine parameters were measured in the samples and their deviation and the linear equation between the level of hemolysis and the deviation were determined. The following threshold values of hemolysis were determined, which correspond to the acceptable analytical (lower value) and clinical (upper value) levels of parameter variability: BHB 0.96 and 4.81; NEFA 0.39 and 3.31; GLU 0.38 and 3.90; ALB 1.12 and 6.11; TPROT 1.40 and 6.80; UREA 6.62 and 20.1; TBIL 0.75 and 5.65; AST 0.11 and 2.18; GGT 1.71 and 8.90, LDH 0.01 and 0.11, ALP 0.97 and 2.95; TGC 1.56 and 15.5; CHOL 1.29 and 8.56; Ca 5.68 and 25.7; P 0.57 and 8.43; Mg 1.10 and 8.47; INS 1.15 and 3.89; T3 8.19 and 15.6; T4 8.97 and 18.5; and CORT 2.78 and 11.22 g/L cell-free hemoglobin. Three decision levels are available for each metabolic and endocrine parameter: if hemolysis is below the lower (analytical) threshold value, results can be reported without restriction; if hemolysis is between the lower and upper thresholds, the results can be issued with guidance in the form of corrective linear equations; and if hemolysis is above the upper (clinical) threshold, the results and sample must be discarded. This method contributes to an optimal approach to hemolysis interference with metabolic profile parameters in blood samples from cows.

## 1. Introduction

The first scientific papers on metabolic parameters in cattle were published more than 100 years ago, and since then the blood metabolite assessment has been an indispensable tool for evaluating the health and productivity of cows [1]. The possibility of applying a metabolic profile that implies the simultaneous determination of a number of blood parameters in cows was described in the early seventies [2].

The determination of biochemical and endocrinological parameters is of irreplaceable importance in research and practice for at least two reasons: (a) a clear connection has been established in cows between the metabolism of polysaccharides, proteins, and lipids and measurable blood parameters, from the nutritional level to the monomer level in the anabolic and catabolic process, and (b) high milk production and high nutritional requirements of cows lead to an increase in subclinical and clinical metabolic disorders that can only be diagnosed by analyzing the cows’ blood metabolic parameters using metabolic profiles [3,4,5,6].

A comprehensive metabolic profile includes parameters measured in the blood in order to determine energy metabolism (glucose, nonesterified fatty acids—NEFA, and beta-hydroxybutyrate—BHB), protein metabolism (total protein, albumin, and urea), mineral metabolism (Ca, P, and Mg), functional status of hepatocytes (aspartate aminotrasferase—AST, gamma-glutamyl transferase—GGT, alkaline phosphatase—ALP, total bilirubin—T.Bil, cholesterol, and triglycerides), and hormonal profile related to metabolism (insulin, cortisol, triiodothyronine—T3, and thyroxine—T4) [7,8]. Modern veterinary medicine recommends routine testing of the metabolic profile during the four seasons and in cows in different periods of lactation, as well as when dietary or certain conditional changes occur on the farm or when a disease is suspected. It is recommended to take as many individual samples as possible, especially frequently around calving [9,10].

The blood from cows is usually sampled at their dwelling place on farms or in pastures and subsequently transported to laboratories where the samples are further processed and prepared for analysis. This phase is called the preanalytical phase, and a variety of factors can affect the value of the parameters tested in the sample during this phase. Most preanalytical factors, such as poor selection of the vacuum tube/anticoagulant or venipuncture needle, inadequate filling of the vacuum tube, high or very low transport temperature and long transport, or inadequate separation of the serum in the laboratory will lead to erythrocyte damage and hemolysis [11]. Hemolysis is the result of the adverse impact of preanalytical factors, and directly affects the values of biochemical and endocrinological parameters in the blood in the form of an increase or decrease in their values, which leads to further errors during interpretation. In a hemolyzed sample, blood parameters deviate from the true value due to spectrophotometric interference, chemical interference, the release of intracellular substances from the erythrocytes or dilution of the sample [12]. In addition, hemolysis also affects the stability of blood parameters in already-separated serum, so the preservation of hemolyzed samples is a problematic procedure with limitations [13].

Hemolysis is detected via visual assessment of the sample (which is discouraged) or by determining the cell-free hemoglobin concentration as an independent index of hemolysis (H-index), and if the level of hemolysis in the sample is high, the sample must be discarded [12,14]. Discarding a cow’s blood samples is particularly complex as it requires redeployment of human and material resources on the farm for blood sampling and re-transportation of the sample, which consequently reduces operational efficiency and increases costs. By using the cell-free hemoglobin concentration (H-index), it is possible to make the following decisions: (1) if the level of hemolysis is such that the bias caused by hemolysis does not exceed the allowable analytical variability (CVa), the results obtained can be freely issued to the user; (2) if the level of hemolysis is such that the bias exceeds the analytical variability but is within the allowable intraindividual variability (reference change value, RCV), the report can be issued with the note that “hemolysis could affect the value of the parameter” with the possible use of corrective formulas; (3) if the level of hemolysis exceeds the intraindividual variability, the results sensitive to hemolysis will be suppressed; and (4) if the level of hemolysis is so high that there is more than 10 g/L of cell-free hemoglobin, the sample must be discarded without analysis and a new sample must be taken [15].

To make the aforementioned decisions, it is necessary to examine the linear relationship between cell-free hemoglobin concentration and the variability of selected biochemical and endocrinological parameters of the metabolic profile, which are expressed as CVa and RCV. These linear relationships must be determined separately for each parameter and are presented on the interferogram, where the value of the cell-free hemoglobin concentration is on the *X*-axis, and the variability of the parameter (with CVa and RCV values plotted) is on the *Y*-axis [16]. By plotting the regression line derived from the interference experiment, one can easily determine the hemolysis level above which the values of CVa and RCV for each analyte separately are exceeded and make an informed judgement about the sample. 

The aim of this research is to determine the aforementioned linear relationships between the level of hemolysis and the variability of blood parameters through a controlled interference experiment. Another goal is to demonstrate the variability and the hemolysis levels on the interferogram and to identify, for each parameter, the hemolysis level that significantly biases the value of an analyte in the sample. Thus, for the first time in dairy cows, it will be determined for each parameter of the metabolic profile which level of hemolysis is considered acceptable, which level of hemolysis requires certain corrective measures, and when it is necessary to reject the results of individual analytes or the sample without further analysis. This will contribute to improving the management of hemolyzed samples and increase the efficiency and profitability of metabolic profiling in cows.

## 2. Materials and Methods

### 2.1. Blood Samples

Blood samples were obtained from 30 healthy cows in early, mid, and late periods of lactation (100–200 days in milk), aged between 2 and 4 years with optimal body condition scores. The blood samples were collected from the coccygeal vein (according to standard procedures: https://www.bristol.ac.uk/vet-school/research/comparative-clinical/veterinary-education/clinical-skills-booklets/cow/ (accessed on 5 March 2024)) and placed in 10 mL heparin collection tubes (Becton, Dickenson and Company, Franklin Lakes, NJ, USA).

### 2.2. Interference Experiment

One of the paired tubes was centrifuged to obtain hemolysis-free samples. Blood from the other tube was passed through the needle 10 times using a syringe to achieve mechanical hemolysis. The mechanical trauma method was preferred to obtain samples similar to the hemolyzed samples sent to the laboratory and containing leukocytes and thrombocytes [12,15]. The tubes were centrifuged at 2000× *g* for 10 min following the manufacturer’s recommendation. In one of the tubes, the cell-free Hb concentration was 0. In the second tube, the cell-free Hb concentration was >10 g/L. The samples with and without hemolysis were diluted, so that samples with a cell-free Hb concentration of 10 g/L were obtained for each cow. Next, we mixed samples with 10 g/L cell-free Hb with samples with 0 g/L to obtain samples with 8, 6, 4, 2, 1, and 0 g/L of cell-free Hb [16]. The hemolyzed samples were diluted with non-hemolyzed samples from the same animal for each of the 30 cows.

### 2.3. Laboratory Analysis 

Analyses were performed for the following biochemical parameters (method/substrate given in parentheses): nonesterified fatty acids, NEFA (acyl-CoA oxidase), beta-hydroxybutyrate, BHB (3-hydroxybutyrate dehydrogenase), glucose, GLU (glucose oxidase), calcium, Ca (Arsenazo III), inorganic phosphates, P (phosphomolybdate), total protein (TPROT), albumin, ALB (green bromocresol), urea, triglycerides (TGC), cholesterol (CHOL), total bilirubin, TBIL (diazo sulfanilic acid), aspartate aminotransferase, AST (2-oxoglutarate, oxalacetate), gamma-glutamyl transferase, GGT (L-γ-glutamyl-3-carboxy-4-nitroanilide/glycylglycine), lactate dehydrogenase LDH (pyruvate reduction), alkaline phosphatase, ALP (4-nitrophenylphosphate), cortisol, CORT (immunoenzyme method), triiodothyronine T3 (immunoenzyme method), and thyroxine T4 (immunoenzyme method) and insulin, INS (immunoenzyme method). Standard kits from Randox (Crumlin, UK) for NEFA and BioSystem (Barcelona, Spain) for other parameters were used on a Rayto Chemray 120 spectrophotometer (Rayto Life and Analytical Sciences, Shenzhen, China). An automated immunoassay analyzer TOSOH AIA-360 (Tosoh Bioscience, Tokyo, Japan) was used for endocrinological analyses.

### 2.4. Statistical Analysis and Interferograms 

The percentage difference was calculated for the samples from each cow. Next, the mean percentage differences were calculated for each Hb concentration using the percentage differences for 30 different cows. The following formula was used: percent difference (%) = (results of hemolyzed sample—result of non-hemolyzed sample)/result of non-hemolyzed sample × 100. An ANOVA analysis with a post hoc LSD test was used to evaluate the difference between the means of blood metabolic and endocrine parameters in seven different groups, divided according to cell-free hemoglobin concentration. The formula for CVa and RCV was used, as in previous experiments [17,18]. Interferograms are presented as graphs, with the concentration of cell-free hemoglobin on the *X* axis, and the percentage of deviation of the parameter values on the *Y* axis. The allowed analytical variability (CVa) and the allowed individual clinical variability (RCV) are presented on the *Y* axis. The parameter values are plotted on the graph in the function of the cell-free hemoglobin concentration. Then, the linear regression equation was determined and the regression line was generated. Based on the intersection of the values on the *X* and *Y* axes of the regression line, it is possible to determine above which level of the sample’s hemolysis an analytically and clinically significant deviation of the parameters will occur in relation to the value in the same sample without hemolysis.

## 3. Results

Table 1 shows the initial concentrations of the analytes as well as the values obtained after dilution. An ANOVA analysis demonstrates substantial deviations in all analyzed parameter values at a statistically significant level (*p* < 0.01) when non-hemolyzed and hemolyzed samples from the same animal are mixed.

The percentage deviation of an analyte concentration in the samples to which cell-free Hb was added compared to the paired sample in which no hemolysis occurred (0%) is shown in Table 2. A positive bias was demonstrated by BHB, NEFA, ALB, TPROT, UREA, AST, LDH, TGC, CHOL, Ca, P, and Mg and negative bias was detected for GLU, TBIL, GGT, ALP, INS, T3, T4, and CORT. The SER for all parameters was between 0.07 and 0.14%. Parameter deviations in the sample with the highest value of cell-free Hb were in the range of 0–10% for UREA, Ca, and Mg; 11–50% for GLU, ALB, TPROT, GGT, ALP, TGC, CHOL, P, T3, T4, and CORT; and 51–100% for BHB, TBIL, INS and >100% for NEFA, AST, and LDH.

Linear equations and their coefficients of determination were established based on the percentage change in analyte concentration and cell-free Hb concentration as independent variables (Table 3). The hemoglobin concentration considerably (*p* < 0.01) impacted the analyte bias reported as a percentage, and for the majority of the analytes undergoing analysis, exceptionally high coefficients of determination R^2^ >0.98 were observed. The degree of hemolysis was calculated using these linear methods, which resulted in a percentage deviation in the parameters that was greater than the analytically and clinically acceptable variations (CVa and RCV). Table 3 displays the hemolysis thresholds determined. The values of the analytical cut-off (CVa) and the clinical cut-off (RCV) were calculated and are presented in this Table. The Hb concentrations corresponding to these values were calculated based on the regression equation (Table 3). 

**Figure 1:**
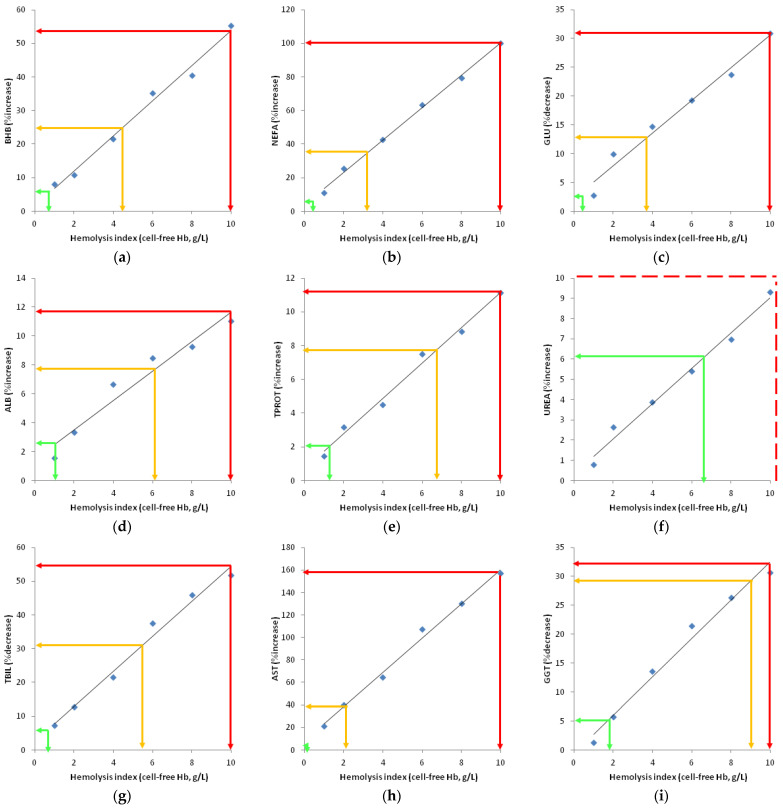

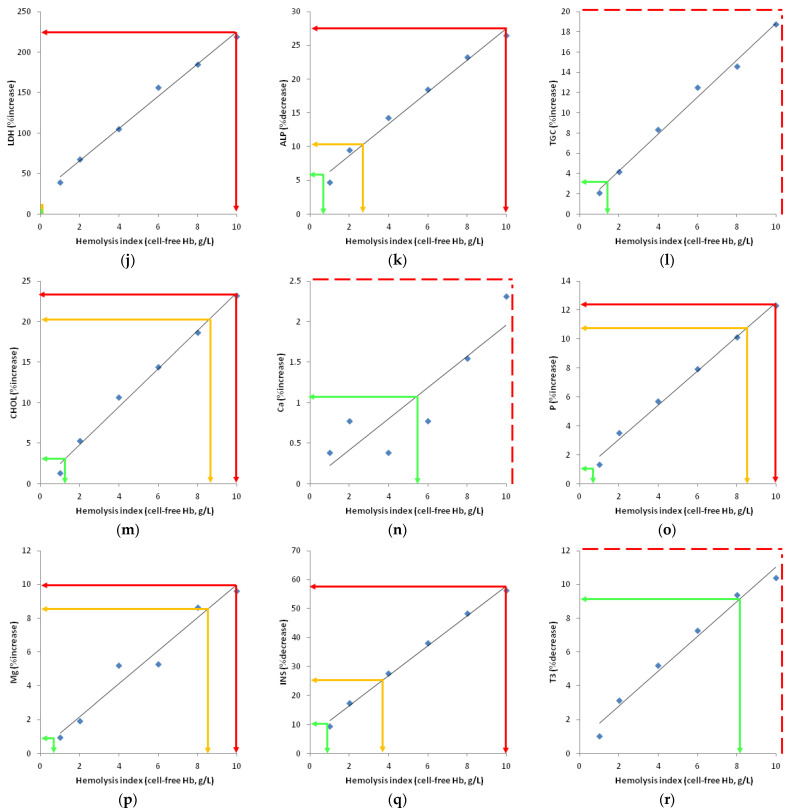

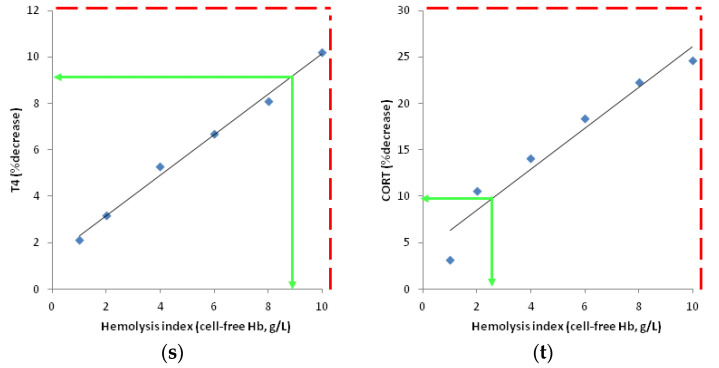
The interferograms of the blood biochemical and endocrine parameters in cows. Green—cut off for analytically important variability, direct reporting of results, yellow—reporting with interpretation, red—cut off for clinically important variability—results should be discarded.

The interferograms of the blood biochemical and endocrine parameters in cows are displayed in Figure 1a–t. A decision regarding the test results obtained, including direct reporting, reporting with interpretation, and rejection of the tested parameters, can be made based on the degree of hemolysis and the deviation in the values.

The parameters of energy metabolism exhibited the following level of sensitivity to hemolysis. When Hb is below 0.96 g/L, the BHB results can be reported directly; when Hb value is between 0.96 and 4.81 g/L, the results can be reported with interpretation (add regression line); and when Hb is above 4.81 g/L, the results should be rejected (Figure 1a). NEFA results can be reported directly for samples with Hb < 0.39 g/L; for Hb 0.39–3.31 g/L, results can be reported with interpretation (add regression line); and for Hb > 3.31 g/L, results should be rejected (Figure 1b). In cases where Hb is below 0.38 g/L, GLU results can be reported directly; if Hb is between 0.38 and 3.90 g/L, the results can be reported with interpretation (add regression line); and if Hb is above 3.90 g/L, the results should be discarded (Figure 1c).

The degree of hemolysis in the sample must be taken into account when reporting the results of the protein status parameters. When Hb is below 1.12 g/L, the ALB results can be reported directly; when the Hb value is between 1.12 and 6.11 g/L, the results can be released with an interpretation (add regression line); and when the Hb value is above 6.11 g/L, the results should be disregarded (Figure 1d). For samples containing Hb < 1.40 g/L, the TPROT results may be issued directly; for samples containing Hb 1.40–6.80 g/L, the results could be reported with an interpretation (add regression line); and for samples containing Hb > 6.80 g/L, the results should be discarded (Figure 1e). For samples containing Hb < 6.62 g/L, UREA results may be reported directly; for samples containing Hb 6.62–20 g/L, results could be issued with interpretation (add regression line) (Figure 1f); and results with Hb > 20 g/L should be discarded according to formula. UREA exhibits a significantly lower sensitivity to hemolysis compared to ALB and TPROT.

It is crucial to report the parameters of the liver’s functional status and the enzyme profile in relation to the degree of hemolysis. The parameters that are most sensitive to hemolysis include LDH and AST, while TGC is the least sensitive. For samples containing Hb <0.75 g/L, TBIL results can be reported directly; for samples containing Hb 0.75–5.65 g/L, the results may be issued with an interpretation (add regression line); and for samples containing Hb >5.65 g/L, the results are appropriate for rejection (Figure 1g). In terms of enzymes, results with Hb (g/L) < 0.11 (AST), <1.71 (GGT), <0.01 (LDH), and <0.97 (ALP) can be reported directly; results with Hb (g/L) 0.11–2.18 (AST), 1.71–8.90 (GGT), 0.01–0.11 (LDH), and 0.97–2.95 (ALP) can be reported with interpretation (add regression line); and the results are appropriate for rejection with Hb (g/L) > 2.18 (AST), >8.90 (GGT), >0.11 (LDH), and <0.97 and >2.95 (ALP) (Figure 1h–k). Regarding the lipid parameters of liver function, data with Hb (g/L) < 1.56 (TGC) and <1.29 (CHOL) could be reported directly; data with Hb (g/L) 1.56–15.5 (TGC) and 1.29–8.56 (CHOL) could be reported with interpretation (add regression line); and data with Hb (g/L) > 15.5 (TGC) and >8.56 (CHOL) should be rejected (Figure 1l,m).

The mineral status of the cows reflected in Ca, P, and Mg concentrations must also be reported regarding the level of hemolysis in the sample. Ca is less sensitive to hemolysis compared to P and Mg. For samples with Hb (g/L) < 5.68 (Ca), <0.57 (P), and <1.10 (Mg), the results can be reported directly; for samples with Hb (g/L) < 5.68–25.7 (Ca), 0.57–8.43 (P), and 1.1–8.47 (Mg), the results could be reported with interpretation (add regression line); and results are appropriate for rejection when Hb (g/L) > 25.7 (Ca), >8.43 (P), and >8.47 (Mg) (Figure 1n–p).

Regarding hormones, insulin exhibits significant sensitivity to hemolysis, while T3, T4, and CORT are analytically but not clinically sensitive to the level of hemolysis (cell-free Hb) in the sample. INS results could be reported directly for samples with Hb < 1.15 g/L; for Hb 1.15–3.89 g/L results could be reported with interpretation (add regression line); and for Hb > 3.89 g/L, results should be rejected (Figure 1q). Concerning other hormones, results could be reported directly for samples with Hb (g/L) < 8.19 (T3), <8.91 (T4), and <2.78 (CORT); results could be issued with interpretation (add regression line) with Hb (g/L) 8.19–15.6 (T3), 8.97–18.5 (T4), and 2.78–11.22 (CORT); and results are appropriate for rejection with Hb (g/L) >15.6 (T3), >18.5 (T4), and >11.22 (CORT) (Figure 1r–t).

## 4. Discussion

### 4.1. Methods of Controlled Hemolysis Interference

The aforementioned study included cows whose endocrine and metabolic parameter values fell within established reference ranges [19,20]. This indicates that the cows in the study had good energy, protein, and mineral status, and their livers were not overburdened. Determining whether in vivo hemolysis occurred in the samples is crucial information to consider before interpreting the results.

In vivo hemolysis is characterized by a parallel increase in LDH and AST values [21], and as the activity of the enzymes mentioned remained within the reference ranges (and there were no hematological signs of in vivo hemolysis—the blood count results), it can be concluded that no individuals or samples with in vivo hemolysis were used in this experiment. Controlled interference experiments such as this one require samples with consistent and non-extreme values of metabolic and endocrine parameters, as susceptibility to hemolysis may depend on the initial concentration of a parameter. Thus, it has been shown that samples with an initially low insulin value or with a high AST value, exhibit a significantly lower sensitivity of these parameters to hemolysis [22,23]. The method used to perform the hemolysis in the experiment affects the response to hemolysis as well.

The most common methods for performing hemolysis in experiments are the addition of pure hemoglobin to the serum pool, osmotic shock, aspiration through a blood collection needle, and freezing/thawing of whole blood. The aspiration method (used in this trial) is considered the most sensitive to hemolysis, while the method involving the addition of cell-free hemoglobin is the least sensitive [24]. Nevertheless, the aspiration method is the closest to real in vitro hemolysis as it reflects the hemolysis that occurs during sampling in the preanalytical phase. In our experiment, we used a sample from the same animal to prepare different dilutions. In mechanically induced hemolysis, the use of a sample from the same animal is crucial because it has a positive effect on the reproducibility of the bias analytes, providing a more realistic representation of the impact of hemolysis in cattle [25].

### 4.2. Variation in Parameter Values as a Result of Hemolysis

Hemolysis led to a decrease in the values of GLU, TBIL, GGT, ALP, and hormones (INS, T3, T4, and CORT), while the other parameters examined showed an increase with increasing intensity of hemolysis. Similar results have been observed in a variety of studies, with some discrepancies possibly attributable to the specifics of the equipment and procedures used as well as the non-standardized presentation of the hemolysis level [26,27,28,29].

Due to the analytes’ significantly lower concentration in erythrocytes than in serum or plasma, there is a dilution effect when the constituents are released from the hemolyzed erythrocytes, which causes the negative bias, or decrease in the values of GLU, TBIL, GGT, and ALP [30]. Furthermore, as cell-free Hb inhibits color development in the colorimetric reaction, a lower TBIL value can also be recorded as a result of chemical interference [31]. At concentrations of 2–5 g/L cell-free Hb, reduced values of various biochemical parameters were observed at an analytically significant level. This is consistent with previous studies that demonstrated visible dilution effects in severe hemolysis > 3 g/L Hb [30]. With respect to hormones, INS showed the highest sensitivity to hemolysis. A significant decrease in its value is due to the release of proteases from the erythrocytes during hemolysis, which destroy peptide hormones such as INS [32]. In addition to the above, hemolysis may interfere with the various methods of biochemical plasma analysis. Hemoglobin can interfere with test reactions, such as bilirubin degradation, which is likely caused by an oxidative reaction involving hydrogen peroxide (produced during hemoglobin oxidation) and acid hematin, which acts as a pseudoperoxidase [33]. A change in the peroxidase activity of the reagent when exposed to cell-free hemoglobin can lead to the degradation of analytes such as GLU or NEFA.

When a hemolyzed sample is added, T3, T4, and CORT exhibit reduced concentrations; nevertheless, a very high level of hemolysis is required to produce a bias that exceeds the analytically or clinically allowable variability of these hormones. CORT and T4 are highly protein-bound hormones in the bloodstream, so the accurate measurement of these hormones depends primarily on the ability of the assay to separate the hormone molecules from the binding proteins [34]. Hormone concentration has been reported to be significantly affected by chemical interference caused by proteases that break down hormones, antibodies, and other protein systems crucial for the accurate determination of hormones in an assay. This has been confirmed in our research by using the chemiluminescence method for hormone measurement, where hemolysis has no spectrophotometric interference [12].

An increase in the level of hemolysis results in a positive bias, or an increase in the levels of BHB, NEFA, ALB, TPROT, UREA, AST, LDH, TGC, CHOL, Ca, P, and Mg. The release of cellular components into the sample is one of the mechanisms leading to a positive bias. Analytes such as LDH, AST, and P are particularly sensitive to hemolysis as their concentration in erythrocytes is 40-fold (AST) or over 100-fold (LDH, P) higher in RBC compared to plasma [35,36]. Previous research has revealed an increase in the NEFA concentration value as a consequence of cell-free hemoglobin increasing the absorbance of the sample during end-point reactions [37,38]. The prevention of an increase in the value of NEFA due to hemolysis can be solved by subtracting the adsorbance value of the sample (sample blank) when calculating the results in order to read the value that was created solely as a result of the colorimetric reaction [36,37]. 

The concentration of BHB in relation to the intensity of hemolysis exhibited a positive bias in cattle [26,39] for similar reasons as NEFA. However, in certain assays, BHB demonstrated stability and insensitivity to hemolysis [38]. A false increase in NEFA and BHB values is very significant and must be corrected as it can lead to a false interpretation of energy balance or detection of ketosis [40]. An increase in the values of other parameters may occur as a result of the analytical interference of cell-free Hb [41,42]. Although Ca concentration increases with the level of hemolysis, it exhibits the least linear relationship with hemoglobin concentration, which is consistent with previous studies [26].

### 4.3. Analytical and Clinical Importance of Hemolysis 

The bias values obtained correspond to previous results in cattle and other animal species [22,23,24,25,26,27,28,29,30,31,32,33,34,35,36,37,38,39,40]. In addition to the above, numerous studies have established a linear correlation between the level of hemolysis/hemolysis indices and the concentration of the parameters studied [41,42]. The linear relationship between the initial concentrations of the parameters and the level of hemolysis could not explain the effect of hemolysis on the variation of the parameter. This problem was solved by creating linear formulas that linked the level of hemolysis to the percentage of bias [28]. Such correlations made it possible to determine hemolysis thresholds that would not exceed the permitted level of parameter variation. Hemolysis and the method of its detection depended on the equipment and methods used, and it was necessary to establish universal methods for evaluating the impact of hemolysis on analytes. Interferograms with bias on the *Y* axis and hemoglobin concentration on the *X* axis have been generated for this purpose. The *Y* axis illustrates two significant biases: the clinically significant bias, represented by RCV, and the analytically significant bias, represented by CVa. By matching these biases to the Hb concentration via the regression line obtained, we can infer the final effect of hemolysis on the variability of the parameter. A threefold effect of hemolysis is possible, and our decision depends on the type of the effect: if the level of hemolysis causes a bias that does not exceed CVa, we release the results without restrictions; if the level of hemolysis causes a bias between CVa and RCV, we release results with a comment that sample hemolysis may have a significant impact; if the level of hemolysis causes a bias that exceeds the RCV we do not release the results and suggest resampling. 

The methods and recommendations mentioned correspond to the official recommendations of the European Federation of Clinical Chemistry and Laboratory Medicine (EFLM) Working Group for the Preanalytical Phase (WG-PRE) [15] and were applied as such in our research procedure on samples originating from cattle. CVa, CVi, and RCV were obtained in our experiment from original laboratory measurements in the assessment of internal quality control, which have been monitored for several years and exhibit good stability with only minor variations [18].

A very significant linear correlation (r^2^ > 0.98 for most of the examined parameters) was determined between the level of hemolysis and the bias of the examined parameters. High correlations and regression lines support the author’s previous results [16,43,44] and provide a framework for the use of interferograms to evaluate the effect of hemolysis on the development of bias. The concentration of cell-free Hb at which an analytically and clinically significant bias occurs is the lowest in the case of LDH and AST. LDH exhibits a clinically significant bias at concentrations of cell-free Hb = 0.11 g/L. High sensitivity has also been confirmed in other experiments where an increase in the LDH value was established at a concentration of Hb = 0.25 g/L to 0.5 g/L [16,42,43]. Clinically significant deviations of AST due to hemolysis occur at a concentration of Hb = 2.18, while other studies have reported that hemolysis has a clinically significant effect on AST at Hb = 0.8–2 g/L [16,43,44].

At hemoglobin concentrations of 0–1.5 g/L, the sample may exhibit signs of a slight yellow or orange discoloration. Macroscopically, this does not indicate hemolysis but may show significant interference with LDH and AST. Regarding the other parameters, their sensitivity to cell-free Hb is generally consistent with previously observed results [44], meaning that a clinically significant bias occurs at Hb concentrations of 2 to 9 g/L; however, these values are higher for triglycerides, as also confirmed by our experiment.

The same source found INS to be much more sensitive to hemolysis than other hormones, which is consistent with our results. When samples with visible hemolysis (cell-free Hb > 2 g/L) are encountered in daily work, it is important to handle them rationally because we can provide results for a wide range of parameters even under such circumstances [29]. In such situations, corrected linear formulas can be provided with a comment on the results to adjust the value obtained, presuming hemolysis has not resulted in a clinically significant bias. For INS, the use of correction formulas to modify results in hemolyzed samples is considered appropriate; however, due to the significant intraindividual variation, this approach may result in inaccuracies for other parameters [45,46].

The rational use of correction formulas in bovine hemolytic samples has not been investigated so far and presents a topic for future research. According to the recommendations of the Clinical and Laboratory Standards Institute (CLSI), it is necessary to regularly validate hemolysis indices due to the potential clinical impact of hemolysis on the results obtained [14]. In our veterinary clinical laboratory that analyzes bovine samples, this study is a step towards the routine validation of hemolysis indices, as recommended by the CSLI.

## 5. Conclusions

The research results demonstrate a significant linear relationship between hemolysis (expressed as cell-free Hb concentration) and analytical and clinical parameter variability (expressed as CVa and RCV), and these relationships can be represented by interferograms and linear equations. By using interferograms and linear equations, it is possible to separately determine the level of hemolysis that corresponds to the significant analytical and clinical interference threshold for each parameter. The results indicate that AST, LDH, NEFA, and INS are most sensitive to hemolysis, while other parameters can be measured and reported using the correction formulas, even in cases of moderate to severe hemolysis. Interferograms and linear equations are the result of simple and easily repeatable methods that can be performed within each laboratory, which will contribute to developing an optimal approach to sample the blood of dairy cows. By eliminating the effect of hemolysis-related interference, these techniques will reduce the number of unnecessary sample discards and improve the interpretation of results.

## Figures and Tables

**Table 1:** Comparison of the initial concentrations of the analytes and the values obtained after dilution using ANOVA.

Blood Parameters Concentration	Cell-Free Hemoglobin Concentration (g/L)							SER	*p*-Value (ANOVA)
	0	1	2	4	6	8	10		
BHB (mmol/L)	0.74 ^a^*	0.8 ^a^	0.82 ^a^	0.9 ^a^	1 ^b^	1.04 ^b^	1.15 ^b^	0.05	0.0012
NEFA (mmol/L)	0.63 ^a^	0.7 ^a^	0.79 ^b^	0.9 ^b^	1.03 ^b^	1.13 ^c^	1.26 ^c^	0.05	0.0009
GLU (mmol/L)	2.91 ^a^	2.83 ^a^	2.62 ^b^	2.48 ^c^	2.35 ^c^	2.22 ^d^	2.01 ^d^	0.14	0.0028
ALB (g/L)	38.9 ^a^	39.5 ^a^	40.2 ^a^	41.5 ^a^	42.2 ^b^	42.5 ^b^	43.2 ^b^	2.35	0.0162
TPROT (g/L)	69.1 ^a^	70.1 ^a^	71.3 ^a^	72.2 ^a^	74.3 ^b^	75.2 ^b^	76.8 ^b^	4.16	0.0182
UREA (mmol/L)	6.45 ^a^	6.5 ^a^	6.62 ^a^	6.7 ^a^	6.8 ^a^	6.9 ^b^	7.05 ^c^	0.38	0.0413
TBIL (μmol/L)	7.02 ^a^	6.51 ^a^	6.12 ^a^	5.51 ^b^	4.38 ^b^	3.8 ^c^	3.39 ^d^	0.30	0.0013
AST (IU/L)	78.6 ^a^	95.5 ^b^	110 ^c^	129.5 ^d^	163.2 ^e^	181.1 ^f^	202.2 ^f^	7.84	0.0002
GGT (IU/L)	22.8 ^a^	22.5 ^a^	21.5 ^a^	19.7 ^b^	17.9 ^c^	16.8 ^d^	15.8 ^e^	1.12	0.0057
LDH (IU/L)	351 ^a^	490 ^b^	590 ^c^	720 ^d^	900 ^e^	1000 ^f^	1120.0 ^g^	42.21	0.0001
ALP (IU/L)	189 ^a^	180 ^a^	171 ^a^	162 ^b^	154 ^c^	145 ^d^	139 ^d^	9.31	0.0056
TGC (mmol/L)	0.24 ^a^	0.245 ^a^	0.25 ^a^	0.26 ^b^	0.27 ^c^	0.275 ^c^	0.285 ^d^	0.01	0.0072
CHOL(mmol/L)	3.75 ^a^	3.8 ^a^	3.95 ^a^	4.15 ^b^	4.29 ^b^	4.45 ^c^	4.62 ^d^	0.24	0.0132
Ca (mmol/L)	2.59 ^a^	2.59 ^a^	2.6 ^a^	2.6 ^a^	2.61 ^a^	2.63 ^b^	2.65 ^b^	0.15	0.0357
P (mmol/L)	2.27 ^a^	2.3 ^a^	2.35 ^a^	2.4 ^b^	2.45 ^b^	2.5 ^c^	2.55 ^c^	0.14	0.0044
Mg (mmol/L)	1.04 ^a^	1.05 ^a^	1.06 ^a^	1.094 ^b^	1.095 ^b^	1.13 ^c^	1.14 ^c^	0.06	0.0223
INS	8.71 ^a^	7.9 ^a^	7.2 ^b^	6.3 ^c^	5.4 ^d^	4.5 ^e^	3.80 ^f^	0.36	0.0016
T3	0.96 ^a^	0.95 ^a^	0.93 ^a^	0.91 ^a^	0.89 ^b^	0.87 ^b^	0.86 ^b^	0.05	0.0123
T4	28.4 ^a^	27.8 ^a^	27.5^a^	26.9 ^b^	26.5 ^b^	26.1 ^b^	25.50 ^c^	1.54	0.0250
CORT	25.6 ^a^	24.8 ^a^	22.9 ^b^	22 ^b^	20.9 ^c^	19.9 ^c^	19.30 ^c^	1.27	0.0011

* different superscript letter means significant difference between group at minimal level *p* < 0.05.

**Table 2:** The percentage deviation (absolute values) of an analyte concentration in the samples to which cell-free Hb was added compared to the paired sample in which no hemolysis occurred (0%).

Blood Parameters (% Bias)	Cell-Free Hemoglobin Concentration (g/L)						
	0	1	2	4	6	8	10
BHB	0.00	8.11	10.81	21.62	35.14	40.54	55.41
NEFA	0.00	11.11	25.40	42.86	63.49	79.37	100.05
GLU	0.00	−2.75	−9.97	−14.78	−19.24	−23.71	−30.93
ALB	0.00	1.54	3.34	6.68	8.48	9.25	11.05
TPROT	0.00	1.45	3.18	4.49	7.53	8.83	11.14
UREA	0.00	0.78	2.64	3.88	5.43	6.98	9.30
TBIL	0.00	−7.26	−12.82	−21.51	−37.61	−45.87	−51.71
AST	0.00	21.50	39.95	64.76	107.63	130.41	157.25
GGT	0.00	−1.32	−5.70	−13.60	−21.49	−26.32	−30.70
LDH	0.00	39.60	68.09	105.13	156.41	184.90	219.09
ALP	0.00	−4.76	−9.52	−14.29	−18.52	−23.28	−26.46
TGC	0.00	2.08	4.17	8.33	12.50	14.58	18.75
CHOL	0.00	1.33	5.33	10.67	14.40	18.67	23.20
Ca	0.00	0.39	0.77	0.39	0.77	1.54	2.32
P	0.00	1.32	3.52	5.73	7.93	10.13	12.33
Mg	0.00	0.96	1.92	5.19	5.29	8.65	9.62
INS	0.00	−9.30	−17.34	−27.67	−38.00	−48.34	−56.37
T3	0.00	−1.04	−3.12	−5.21	−7.29	−9.38	−10.42
T4	0.00	−2.11	−3.17	−5.28	−6.69	−8.10	−10.21
CORT	0.00	−3.13	−10.55	−14.06	−18.36	−22.27	−24.61

**Table 3:** Linear equations and their coefficients of determination based on the percentage change in analyte concentration and cell-free Hb concentration as independent variables.

Blood Parameters	Regression Equation (y-% Bias, x-Hb Conc.)	R^2^	CVa%	HI Cut-off CVa%	RCV	HI Cut-off RCV
BHB	y = 5.3201 x + 0.957	0.9913	6.15	0.96	26.5	4.81
NEFA	y = 9.8218x + 2.5353	0.9972	6.42	0.39	35.1	3.31
GLU	y = −2.9538x − 1.401	0.9794	2.55	0.38	12.9	3.90
ALB	y = 1.0954x + 0.9147	0.9762	2.21	1.12	7.6	6.11
TPROT	y = 1.0849x + 0.426	0.9893	2.08	1.40	7.8	6.80
UREA	y = 0.8905x + 0.1979	0.9892	6.1	6.62	24.5	>10 (20.1)
TBIL	y = −5.3246x − 1.6739	0.9861	5.82	0.75	31.75	5.65
AST	y = 15.678x + 5.0673	0.9935	5.62	0.11	39.2	2.18
GGT	y = −3.2805x + 0.3675	0.9874	5.25	1.71	28.9	8.90
LDH	y = 21.27x + 16.265	0.9843	6.12	0.01	11.6	0.11
ALP	y = −2.557x − 2.5083	0.9801	5.89	0.97	10.1	2.95
TGC	y = 1.8558x + 0.4124	0.9936	3.29	1.56	23.6	>10 (15.5)
CHOL	y = 2.3468x + 0.1215	0.9932	3.14	1.29	20.2	8.56
Ca	y = 0.1944x + 0.0217	0.8343	1.13	5.68	10.8	>10 (25.7)
P	y = 1.2163x + 0.4661	0.9919	1.15	0.57	10.7	8.43
Mg	y = 0.9866x + 0.1498	0.9709	1.23	1.10	8.5	8.47
INS	y = −5.5017x − 3.7803	0.9875	10.1	1.15	25.2	3.89
T3	y = −1.0701x − 0.4693	0.9816	9.22	8.19	16.8	>10 (15.6)
T4	y = −0.9452x − 0.8947	0.9797	9.31	8.91	17.5	>10 (18.5)
CORT	y = −2.4264x − 2.5357	0.9434	9.28	2.78	27.1	>10 (11.22)

## Data Availability

The data presented in this study are available in the article.

## References

[1] Overton T.R., McArt J.A.A., Nydam D.V. (2017). A 100-Year Review: Metabolic health indicators and management of dairy cattle. J. Dairy Sci..

[2] Payne J.M. (1972). The compton metabolic profile test. Proc. R. Soc. Med..

[3] Arias-Islas E., Morales-Barrera J., Prado-Rebolledo O., García-Casillas A. (2020). Metabolism in ruminants and its association with blood biochemical analytes. Abanico Vet..

[4] Tufarelli V., Puvača N., Glamočić D., Pugliese G., Colonna M.A. (2024). The Most Important Metabolic Diseases in Dairy Cattle during the Transition Period. Animals.

[5] Pascottini O.B., Leroy J.L.M.R., Opsomer G. (2020). Metabolic Stress in the Transition Period of Dairy Cows: Focusing on the Prepartum Period. Animals.

[6] Ruprechter G., de Lourdes Adrien M., Larriestra A., Meotti O., Batista C., Meikle A., Noro M. (2018). Metabolic predictors of peri-partum diseases and their association with parity in dairy cows. Res. Vet. Sci..

[7] Krnjaić S., Cincović M., Djoković R., Belić B., Ježek J., Starič J. (2022). The Influence of Energy Balance, Lipolysis and Ketogenesis on Metabolic Adaptation in Cows Milked Twice and Three Times Daily. Metabolites.

[8] Van Saun R.J. (2023). Metabolic profiling in ruminant diagnostics. Vet. Clin. Food Anim. Pract..

[9] Puppel K., Kuczyńska B. (2016). Metabolic profiles of cow’s blood; a review. J. Sci. Food Agric..

[10] Kabir M., Hasan M.M., Tanni N.S., Parvin M.S., Asaduzzaman M., Ehsan M.A., Islam M.T. (2022). Metabolic profiling in periparturient dairy cows and its relation with metabolic diseases. BMC Res. Notes.

[11] Humann-Ziehank E., Ganter M. (2012). Pre-analytical factors affecting the results of laboratory blood analyses in farm animal veterinary diagnostics. Animal.

[12] Simundic A.M., Baird G., Cadamuro J., Costelloe S.J., Lippi G. (2020). Managing hemolyzed samples in clinical laboratories. Crit. Rev. Clin. Lab. Sci..

[13] Kovačević D., Cincović M., Belić B., Đoković R., Majkić M. (2021). Blood serum stability limit and maximum storage time of bovine samples. Acta Sci. Vet..

[14] (2012). Hemolysis, Icterus, and Lipemia/Turbidity Indices as Indicators of Interference in Clinical. Laboratory Analysis—Approved Guideline.

[15] Lippi G., Cadamuro J., von Meyer A., Simundic A.M., European Federation of Clinical Chemistry and Laboratory Medicine (EFLM) Working Group for Preanalytical Phase (WG-PRE) (2018). Practical recommendations for managing hemolyzed samples in clinical chemistry testing. Clin. Chem. Lab. Med. (CCLM).

[16] Uçar K.T., Çat A., Gümüş A., Nurlu N. (2022). Interferograms plotted with reference change value (RCV) may facilitate the management of hemolyzed samples. J. Med. Biochem..

[17] Freeman K.P., Baral R.M., Dhand N.K., Nielsen S.S., Jensen A.L. (2017). Recommendations for designing and conducting veterinary clinical pathology biologic variation studies. Vet. Clin. Pathol..

[18] Kovačević V., Cincović M.R., Belić B., Djoković R., Lakić I., Radinović M., Potkonjak A. (2021). Biological variations of hematologic and biochemical parameters in cows during early lactation. Pol. J. Vet. Sci..

[19] Cozzi G., Ravarotto L., Gottardo F., Stefani A.L., Contiero B., Moro L., Brscic M., Dalvit P. (2011). Reference values for blood parameters in Holstein dairy cows: Effects of parity, stage of lactation, and season of production. J. Dairy Sci..

[20] Nozad S., Ramin A.G., Moghaddam G., Asri-Rezaei S., Kalantary L. (2014). Monthly evaluation of blood hematological, biochemical, mineral, and enzyme parameters during the lactation period in Holstein dairy cows. Comp. Clin. Pathol..

[21] Lippi G., Cervellin G., Favaloro E.J., Plebani M. (2012). In Vitro and In Vivo Hemolysis: An Unresolved Dispute in Laboratory Medicine.

[22] Durmaz M., Erguder I. (2022). Evaluation of hemolysis interference and possible protective effect of N-phenyl maleimide on the measurement of small peptides. Turk. J. Biochem..

[23] Liu S., Li J., Ning L., Wu D., Wei D. (2021). Assessing the influence of true hemolysis occurring in patient samples on emergency clinical biochemistry tests results using the VITROS^®^ 5600 Integrated system. Biomed. Rep..

[24] Nikolac Gabaj N., Miler M., Vrtaric A., Celap I., Bocan M., Filipi P., Radisic Biljak V., Simundic A., Supak Smolcic V., Kocijancic M. (2022). Comparison of three different protocols for obtaining hemolysis. Clin. Chem. Lab. Med. (CCLM).

[25] Li Z., Hu J., Kamberi M., Rapoza R.J. (2023). Mechanical stress-induced hemolysis of bovine blood is donor-dependent. Artif. Organs.

[26] Jacobs R.M., Lumsden J.H., Grift E. (1992). Effects of bilirubinemia, hemolysis, and lipemia on clinical chemistry analytes in bovine, canine, equine, and feline sera. Can. Vet. J..

[27] Lippi G., Luca Salvagno G., Montagnana M., Brocco G., Cesare Guidi G. (2006). Influence of hemolysis on routine clinical chemistry testing. Clin. Chem. Lab. Med. (CCLM).

[28] Du Z., Liu J., Zhang H., Bao B., Zhao R., Jin Y. (2019). Determination of hemolysis index thresholds for biochemical tests on Siemens Advia 2400 chemistry analyzer. J. Clin. Lab. Anal..

[29] Larrán B., López-Alonso M., Miranda M., Graña A., Rigueira L., Orjales I. (2024). Influence of haemolysis on blood biochemistry profiles in cattle. Res. Vet. Sci..

[30] Koseoglu M., Hur A., Atay A., Cuhadar S. (2011). Effects of hemolysis interferences on routine biochemistry parameters. Biochem. Medica.

[31] van der Woerd-de Lange J.A., Guder W.G., Schleicher E., Paetzke I., Schleithoff M., Wieland O.H. (1983). Studies on the interference by haemoglobin in the determination of bilirubin. J. Clin. Chem. Clin. Biochem..

[32] Lippi G., Blanckaert N., Bonini P., Green S., Kitchen S., Palicka V., Vassault A.J., Plebani M. (2008). Haemolysis: An overview of the leading cause of unsuitable specimens in clinical laboratories. Clin. Chem. Lab. Med..

[33] Shull B.C., Lees H., Li P.K. (1980). Mechanism of interference by hemoglobin in the determination of total bilirubin. I. Method of Malloy-Evelyn. Clin. Chem..

[34] Ward G., Simpson A., Boscato L., Hickman P.E. (2017). The investigation of interferences in immunoassay. Clin. Biochem..

[35] Kirschbaumweg L.T. (2002). Hemolysis as influence & interference factor. J. Int. Fed. Clin. Chem. Lab. Med..

[36] Pai S.H., Cyr-Manthey M. (1991). Effects of hemolysis on chemistry tests. Lab. Med..

[37] Morris J.D., Fernandez J.M., Chapa A.M., Gentry L.R., Thorn K.E., Weick T.M. (2002). Effects of sample handling, processing, storage and hemolysis on measurements of key energy metabolites in ovine blood. Small Rumin. Res..

[38] Stokol T., Nydam D.V. (2006). Effect of hemolysis on nonesterified fatty acid and beta-hydroxybutyrate concentrations in bovine blood. J. Vet. Diagn. Investig..

[39] Larsen T., Nielsen N.I. (2005). Fluorometric determination of beta-hydroxybutyrate in milk and blood plasma. J. Dairy Sci..

[40] Couperus A.M., Schroeder F., Hettegger P., Huber J., Wittek T., Peham J.R. (2021). Longitudinal metabolic biomarker profile of hyperketonemic cows from dry-off to peak lactation and identification of prognostic classifiers. Animals.

[41] Yücel D., Dalva C. (1992). Effect of in vitro hemolysis on 25 common biochemical tests. Clin. Chem..

[42] Di Martino G., Stefani A.L., Lippi G., Gagliazzo L., McCormick W., Gabai G., Bonfanti L. (2015). The degree of acceptability of swine blood values at increasing levels of hemolysis evaluated through visual inspection versus automated quantification. J. Vet. Diagn. Investig..

[43] Marques-Garcia F., Jung D.H.H., Pérez S.E. (2022). Impact of individualized hemolysis management based on biological variation cut-offs in a clinical laboratory. Ann. Lab. Med..

[44] Douhal Y., Zamorano B., Rivas Chacón L.D.M., Cuadrado Galván E. (2020). Study of Interference Produced by Haemolysis in 73 Analytical Tests. Biomed. J. Sci. Tech. Res..

[45] Wu Z.Q., Lu J., Chen H., Chen W., Xu H.G. (2017). Individualized correction of insulin measurement in hemolyzed serum samples. Immunol. Res..

[46] Lippi G., Avanzini P., Pavesi F., Bardi M., Ippolito L., Aloe R., Favaloro E.J. (2011). Studies on in vitro hemolysis and utility of corrective formulas for reporting results on hemolyzed specimens. Biochem. Medica.

